# Resveratrol attenuates cigarette smoke induced endothelial apoptosis by activating Notch1 signaling mediated autophagy

**DOI:** 10.1186/s12931-021-01620-3

**Published:** 2021-01-19

**Authors:** Dan-dan Zong, Xiang-ming Liu, Jin-hua Li, Ruo-yun Ouyang, Ying-jiao Long, Ping Chen, Yan Chen

**Affiliations:** 1 Department of Respiratory and Critical Care Medicine, The Second Xiangya Hospital, Central South University, Changsha, 410011 Hunan China; 2 Research Unit of Respiratory Disease, Central South University, Changsha, 410011 Hunan China; 3 Diagnosis and Treatment Center of Respiratory Disease, Central South University, Changsha, 410011 Hunan China

**Keywords:** Resveratrol, Notch1 signaling, Endothelial cells, Apoptosis, Autophagy

## Abstract

**Background:**

Increasing evidence shows that endothelial apoptosis contributes to cigarette smoke (CS)-induced disease progression, such as chronic obstructive pulmonary disease (COPD). Our previous studies have validated Notch1 as an anti-apoptotic signaling in CS-induced endothelial apoptosis. Resveratrol (RESV) is a naturally occurring polyphenol that exhibits an anti-apoptotic activity in endothelial cells that exposed to many kinds of destructive stimulus. However, the effects of resveratrol on Notch1 signaling in CS-induced endothelial apoptosis have not yet been fully elucidated. Therefore, the aim of this study was to examine whether RESV can protect endothelial cells from CS-induced apoptosis via regulating Notch1 signaling.

**Methods:**

Human umbilical vein endothelial cells (HUVECs) were pretreated with RESV for 2 h, followed by cotreatment with 2.5%CSE for 24 h to explore the role of RESV in CSE induced endothelial apoptosis. 3-methyladenine (3-MA) or rapamycin was used to alter autophagic levels. Lentivirus Notch1 intracellular domain (LV-N1ICD), γ-secretase inhibitor (DAPT) and Notch1 siRNA were used to change Notch1 expression. The expression of Notch1, autophagic and apoptotic markers were examined by Western blot and the apoptosis rate was detected by Flow cytometry analysis.

**Results:**

Our results showed that activating autophagy reduced CSE-induced endothelial apoptosis, while blocking autophagy promoted cell apoptosis in HUVECs. RESV pretreatment attenuated the CSE-induced endothelial apoptosis and activated Notch1 signaling. RESV pretreatment also increased LC3b-II and Beclin1 production, decreased p62 and mTOR expression. 3-MA treatment inhibited autophagy and aggravated CSE induced apoptosis, while rapamycin promoted autophagy, led to a decrease in cell apoptosis. LV-N1ICD transfection upregulated autophagy and reduced apoptosis. However, this protective effect was abolished by 3-MA treatment. In cells treated with DAPT or Notch1 siRNA, autophagy was decreased, while apoptosis was increased. RESV partly rescued the DAPT or Notch1 siRNA induced apoptosis by activating Notch1 signaling.

**Conclusion:**

In HUVECs, RESV attenuates CSE induced endothelial apoptosis by inducing autophagy in a Notch1-dependent manner.

## Background

Cigarette smoke (CS) is well known to be a predominant risk factor for the development and progression of many diseases, including chronic obstructive pulmonary disease (COPD), coronary heart disease, cerebrovascular disease and cancers. Increasing evidence demonstrated that endothelial apoptosis serves a key role in the pathogenesis of CS related diseases [1]. Our previous studies confirmed that cigarette smoke extract (CSE) can induce endothelial apoptosis in dose- and time-dependent manner [2–6]. Moreover, increased endothelial apoptosis was found in the lungs of CSE induced emphysema mice, and preventing endothelial apoptosis could partly reverse the pathological changes of emphysema [7, 8]. Thus, protecting endothelial cells against CS induced apoptosis is vital to reverse disease progression.

Resveratrol (3,4′,5-trihydroxystilbene; RESV), a natural polyphenol phytoalexin isolated from a variety of plant species, is a well-recognized anti-inflammatory, anti-oxidant, anti-aging and cancer chemopreventive agent. A growing body of evidence suggests that RESV may delay the onset of a number of diseases, including cardiovascular disease, diabetes, cancer and neurodegenerative diseases [9]. RESV was confirmed to exert anti-apoptotic effects, which protect the endothelial cells against the adverse effects of CS-induced oxidative stress [10]. Nevertheless, the underlying mechanisms remain uncharacterized. Autophagy is a fundamental intracellular process, which can degrade microorganisms (viruses, bacteria, fungi and protists/protozoa) and damaged organelles and proteins that cannot be degraded by the proteasome, providing raw materials for cell reconstruction, regeneration, and repair, thereby ensuring the metabolic balance of cells. Furthermore, RESV has been found to exert a protective effect via regulating autophagy [11]. However, no study has focused on the role of RESV-induced autophagy in CS-related endothelial apoptosis.

Notch1 is a transmembrane receptor that mediates intracellular signaling involved in cell differentiation, survival, and apoptosis. Work by our group and others has validated the Notch1 pathway as an anti-apoptotic pathway in CS-induced endothelial apoptosis [2, 3]. As reported, RESV has been suggested to induce functional Notch1 protein expression and activated the pathway by transcriptional regulation [12, 13]. Additionally, Notch1 activity in T-regulatory cells was necessary for autophagy induction and cell survival in response to nutritional deprivation [14, 15]. Therefore, we hypothesized that the protective effect of RESV in response to CS-induced endothelial apoptosis occurs via Notch1-mediated autophagy. In the present study, we investigated whether RESV could regulate cell autophagy and apoptosis of human umbilical vein endothelial cells (HUVECs) that exposed to CSE, and whether RESV exerted a protective effect on CSE induced endothelial apoptosis via Notch1 signaling. Furthermore, we explored whether Notch1 signaling could function as an anti-apoptotic factor through regulating the activation of autophagy.

## Methods

### Cell lines and cell culture

HUVECs were obtained from the American Type Cell Culture Collection (ATCC, lot no.: CRL-1730) and cultured in RPMI-1640 medium (Gibco Laboratories, Grand Island, N.Y.) supplemented with 10% fetal calf serum (Gibco) in a humidified, 5% CO2 atmosphere at 37 °C. Cells were passaged at 80% confluence and grown to full confluence for the experiments. Each experiment was performed in triplicate and repeated independently at least three times.

### Preparation of CSE

CSE was prepared as previously described [2]. Briefly, one nonfiltered Fu-Rong cigarette (tar: 12 mg/cigarette; nicotine: 1.1 mg; and carbon monoxide: 14 mg; China Tobacco Hunan Industrial., Changsha, China) was burned, and the smoke was passed through 25 ml of phosphate-buffered saline via a vacuum pump. This 100% CSE solution was adjusted to 7.2 – 7.4 and filtered through a 0.22 m membrane filter to remove large particles and bacteria before use. CSE was freshly prepared within the 30 min preceding each experiment.

### Cell treatment

After serum starvation for 24 h, HUVEC medium was supplemented with 2.5% CSE for 24 h as described previously [6]. To investigate the role of RESV in CSE induced apoptosis, after serum starvation, HUVECs were pretreated with 40 μM RESV (R5010, Sigma-Aldrich Co., St Louis, MO, USA) for 2 h, followed by cotreatment with 2.5% CSE for 24 h. To examine the effects of autophagy on HUVEC apoptosis, the cells were cultured for 2 h in the presence or absence of the potential 5 mM of the autophagy inhibitor 3-methyladenine (3-MA) (A8353, APExBIO, Houston, TX, USA) or 50 nM of the autophagy inducer rapamycin (Rapa) (A8167, APExBIO, Houston, TX, USA) prior to CSE intervention. For Notch1 inhibition, HUVECs were treated with γ-secretase inhibitor DAPT (D5942, Sigma-Aldrich Co., St Louis, MO, USA) for 24 h.

### Flow cytometry analysis

Apoptosis was assessed using an Annexin V-FITC Apoptosis Detection Kit (BD Biosciences, USA). After treatment, cells were harvested and washed twice with ice-cold PBS and binding buffer and incubated with AnnexinV-FITC in dark for at least 15 min. Propidium iodide (PI) was added in the dark for 10 min at room temperature. Apoptosis was determined by flow cytometry (A00-1-1102, Beckman, USA). The degree of early apoptosis was determined as the percentage of cells positive for Annexin V and negative for PI by flow cytometry. Cells staining negative for Annexin V and PI were considered to be normal. Cells staining positive for Annexin V and negative for PI represented early apoptosis. Finally, cells staining positive for both Annexin V and PI were classified as late apoptosis.

### Notch1 lentivirus and siRNA transfection

cDNA encoding a constitutively active form of Notch1 consisting of the intracellular domain (N1ICD, base pairs: 5308–7665; amino acids: 1770-2555) was synthesized and subcloned into the multicloning site of the lentiviral vector pNL-IRES2-EGFP to generate pNL-N1ICD-EGFP by the GeneChem Corporation (Shanghai, China). siRNA duplexes were produced by RiboBio Corporation (Guangzhou, China) against human Notch1. Scrambled control siRNA, which was used as a negative control (NC), was also designed and obtained from RiboBio Corporation. Lentivirus and siRNA transfections were performed using Lipofectamine 3000 (ThermoFisher Scientific, USA) following the manufacturer’s instructions. Cells of each group were collected 48–72 h after transfection. The transfection efficiency of N1ICD was determined by counting the percentage of EGFP positivity cells. In our experimental conditions, the transfection efficiency average reached 75.37% ± 3.21.

### Quantitative real-time polymerase chain reaction (qPCR)

After extraction, RNA was reverse-transcribed using the RevertAid First Strand cDNA Synthesis Kit (Thermo Fisher Scientific). Subsequently, qPCR was performed using a StepOne Plus real-time PCR system (Life Technologies, Carlsbad, CA, USA). All of the primers were obtained from Sangon Biotech (Shanghai, China). All mRNA expression values were presented relative to β-actin and analyzed by the 2^−ΔΔCt^ method. The primers used for qPCR is listed in Table 1.

**Table 1:** Primer sequences for quantitative real-time polymerase chain reaction

Gene	Primer sequence (5′ → 3′)
Notch1	F: CGCCTTTGTGCTTCTGTTCTT
	R: TCCCGCCGCTTCTTCTT
Notch2	F: ATATTGATGACTGCCCTAACCAC
	R: ATCTCCACTCCAGCCGTTG
Notch4	F: CCCATTAAAAGGCAGGCTGGA
	R: CACGTGGAAGATGTCTGCTCT
Jag1	F: TACTGTGGGACTCATCAGCCGTGT
	R: CAGCAATTTCACAGTTGGGTCCT
Jag2	F: TCGTACTTGCACTCACAATACCA
	R: CACACTCGCAGCGGAACCA
Dll1	F: CCAGCCCTTGTAAGAACGGA
	R: ACAGATTTTGCCGTAGAAGCC
Dll4	F: ATGCCTGTGCCTCGAGTCCC
	R: CCAGCCCCACACCCAGCGAGA
β-actin	F: ACCCTGAAGTACCCCATCGAG
	R: AGCACAGCCTGGATAGCAAC

### Western blot analysis

Cells were harvested and digested in a lysis buffer. Total protein concentration was determined by the BCA protein assay kit. Proteins were separated by 10% SDS-PAGE and were transferred onto PVDF membranes, then incubated with antibodies to Notch1 (#3608, Cell Signaling Technology, Danvers, MA, USA), N1ICD (#4147, Cell Signaling Technology, Danvers, MA, USA), cleaved caspase-3 (#9664, Cell Signaling Technology, Danvers, MA, USA), LC3b (14600-1-AP, Proteintech Group, Inc., Rosemont, IL, USA), Beclin1 (11306-1-AP, Proteintech Group, Inc., Rosemont, IL, USA), mammalian target of rapamycin (mTOR) (20657-1-AP, Proteintech Group, Inc., Rosemont, IL, USA), Hes1 (ab108937, Abcam, Cambridge, UK) and P62/SQSTM1 (184201-AP, Proteintech Group, Inc., Rosemont, IL, USA). After washing, membranes were incubated with HRP-conjugated secondary antibody for 1 h. The bound complexes were detected using enhanced chemiluminescence detection system (Santa Cruz Biotechnology, Dallas, TX, USA). β-actin (600008–1, Proteintech Group, Inc., Rosemont, IL, USA) was probed to confirm equal protein loading and transfer.

### Statistical analysis

Quantitative data are presented as the means ± SD. Statistical analysis was performed using a software package (SPSS 21.0, SPSS Inc., Chicago, IL, USA). One-way ANOVA was used for multiple groups to analyze any differences between two groups. Tukey’s test or Dunnett’s T3 test was used for post hoc multiple comparisons according to the homogeneity test of variance. A value of *p* < 0.05 was considered to be statistically significant.

## Results

### Effect of autophagy on CSE-induced endothelial apoptosis

**Fig. 1:**
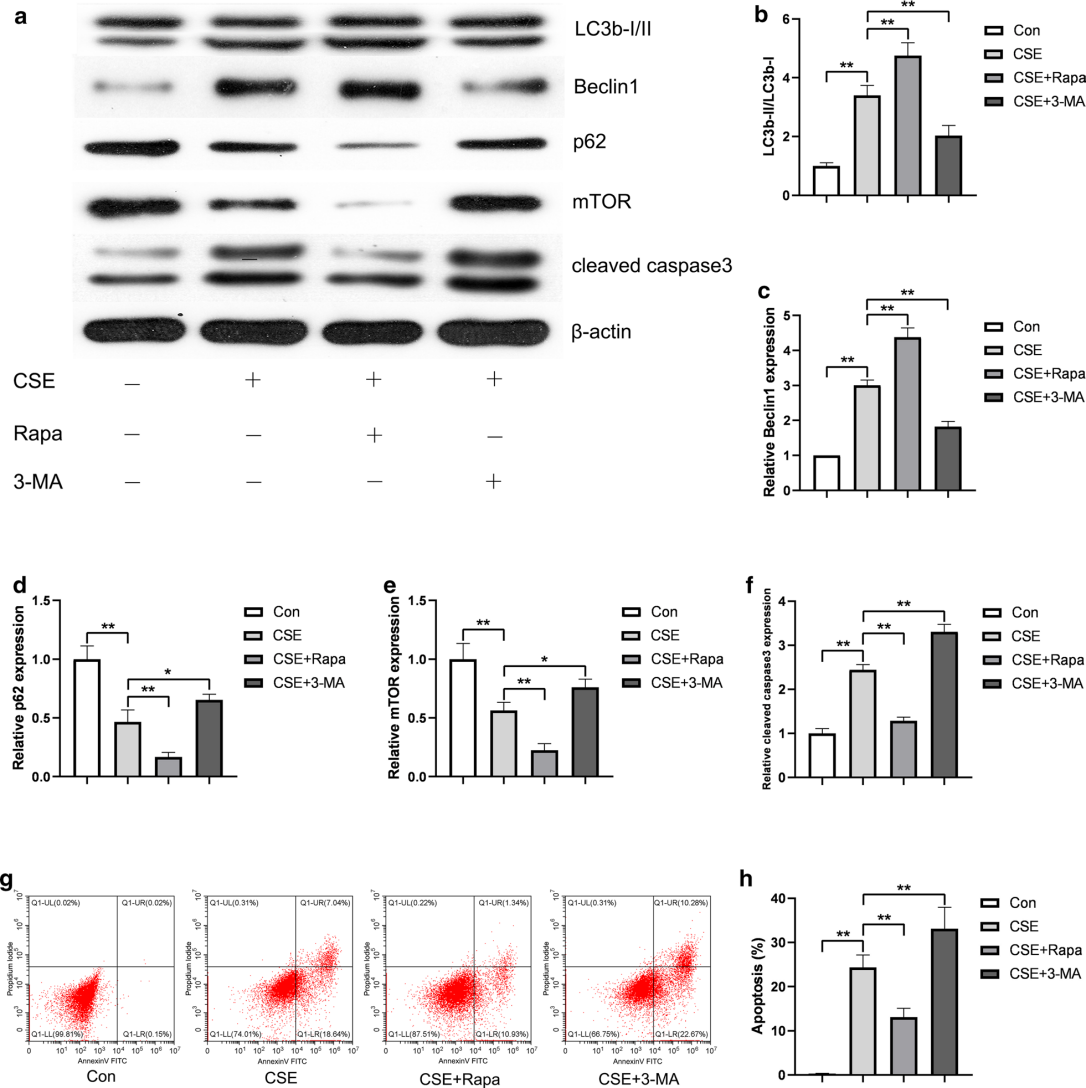
Effect of autophagy on CSE-induced endothelial apoptosis in HUVECs. HUVECs were incubated with 2.5% CSE for 24 h. To examine the effects of autophagy on HUVEC apoptosis, the cells were cultured for 2 h in the presence or absence of 5 mM 3-MA or 50 nM Rapa prior to CSE intervention. Autophagy related proteins (LC3b-I/II, Beclin1, p62, mTOR) and apoptotic protein (cleaved caspase3) were detected by Western blot. β-Actin was used as loading control (**a**). Bar charts show the quantification of LC3B-II/LC3b-I, Beclin1, P62, mTOR and cleaved caspase3. Values are presented as means ± SD (**b**, **c**, **d**, **e**, **f**). Cell apoptosis was detected by flow cytometry (**g**) and statistical analysis was calculated using One-way ANOVA (h). *P < 0.05, **P < 0.01 compared between the marked groups

According to our previous studies, for a high early apoptosis rate and moderate late apoptosis or necrosis rate, a 24 h treatment with 2.5% CSE was selected [6]. For the measure of the autophagic and apoptotic effects of CSE exposure, we assessed the expression level of the Beclin1, LC3B-II, p62, mTOR, cleaved caspase 3 by Western blot and detected the apoptosis rate by flow cytometry. CSE treated HUVECs exhibited a significant increase in the accumulation of LC3B-II (Fig. 1a, b), the expression of Beclin1 (Fig. 1a, c) and cleaved caspase 3 proteins (Fig. 1a, f), as well as the apoptosis rate (Fig. 1g, h). The expression of p62 (Fig. 1a, d) and mTOR (Fig. 1a, e) significantly decreased after CSE stimulation. To detect the role of autophagy on CSE induced apoptosis, we pretreated HUVECs with the rapamycin or 3-MA to promote or inhibit autophagy. The results of LC3B-II, Beclin1, p62 and mTOR expression levels showed significant difference between groups with and without pretreatment of rapamycin and 3-MA (Fig. 1a–e). Meanwhile the pretreatment of rapamycin significantly reduced the cleaved caspase 3 levels and the apoptosis rate induced by CSE. Moreover, pretreated with 3-MA exerted an opposite effect (Fig. 1a, f–h). These data suggested that CSE induced apoptosis of HUVEC could be reversed partly by autophagy in HUVECs.

### RESV stimulates autophagy and protects HUVECs from CSE-induced apoptosis

**Fig. 2:**
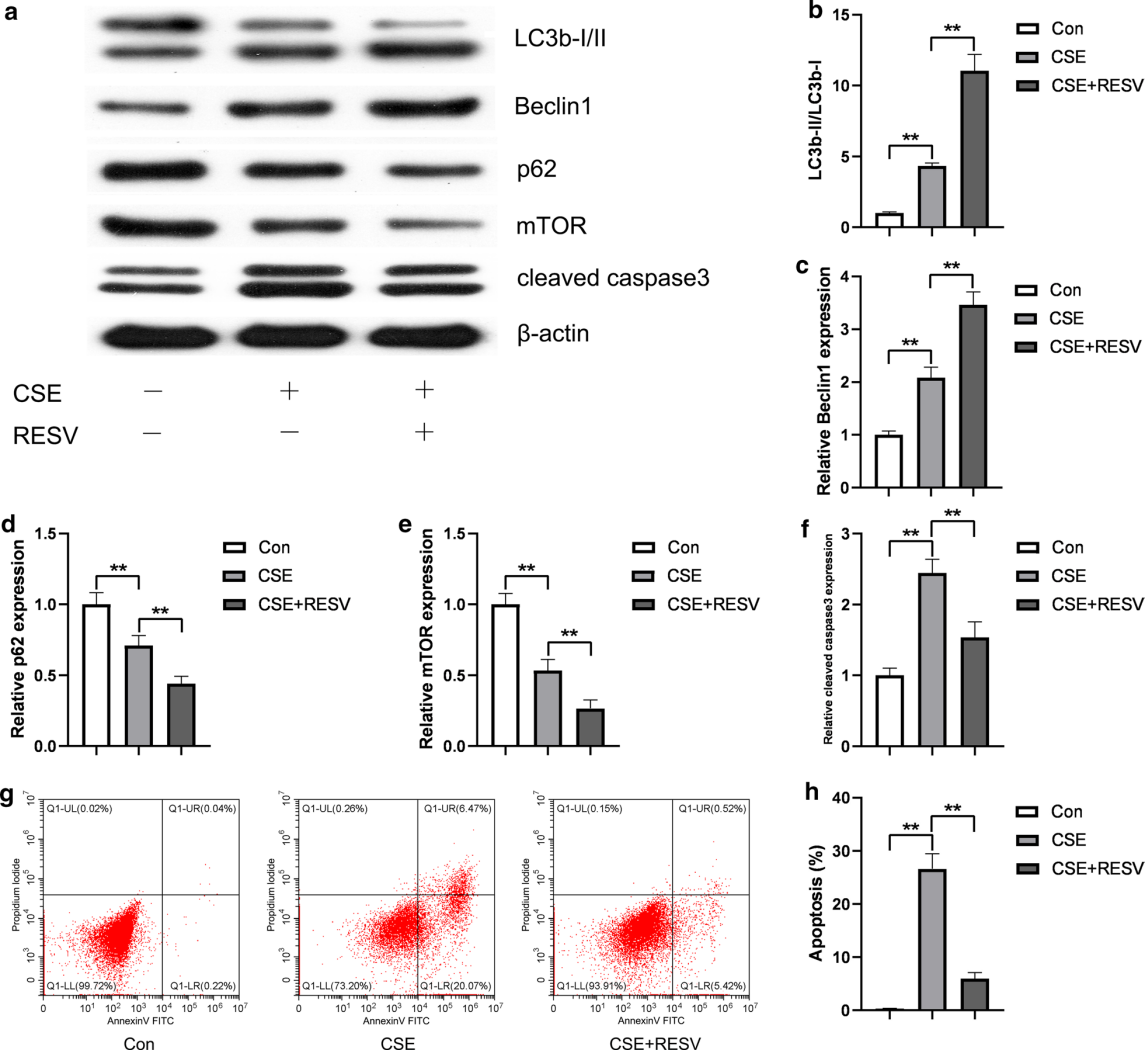
RESV attenuated apoptosis and induced autophagy in CSE treated HUVECs. To investigate the role of RESV in CSE induced apoptosis, HUVECs were pretreated with 40 μM RESV for 2 h, followed by cotreatment with 2.5% CSE for 24 h. Autophagy related proteins (LC3b-I/II, Beclin1, p62, mTOR) and apoptotic protein (cleaved caspase3) were detected by Western blot. β-Actin was used as loading control (**a**). Bar charts show the quantification of LC3B-II/LC3b-I, Beclin1, P62, mTOR and cleaved caspase3. Values are presented as means ± SD (**b**, **c**, **d**, **e**, **f**). Cell apoptosis was detected by flow cytometry (**g**). Statistical analysis was calculated (**h**). *P < 0.05, **P < 0.01 compared between the marked groups

To examine the effect of RESV on cell apoptosis, the cells were pretreated with or without RESV prior to CSE. The LC3B-II and Beclin1 level increased significantly, while the expression level of p62 and mTOR decreased significantly in the RESV and CSE cotreatment group compared to the CSE group (Fig. 2a–e,  *P* < 0.05). In contrast, after combined RESV and CSE treatment, there was a significant decrease in the cleaved caspase 3 expression level (Fig. 2a, f) and a reduction in HUVEC apoptosis rate (Fig. 2g, h). These results indicated that RESV may attenuate CSE-induced apoptosis in HUVECs through activating autophagy.

### RESV influenced Notch signaling

**Fig. 3:**
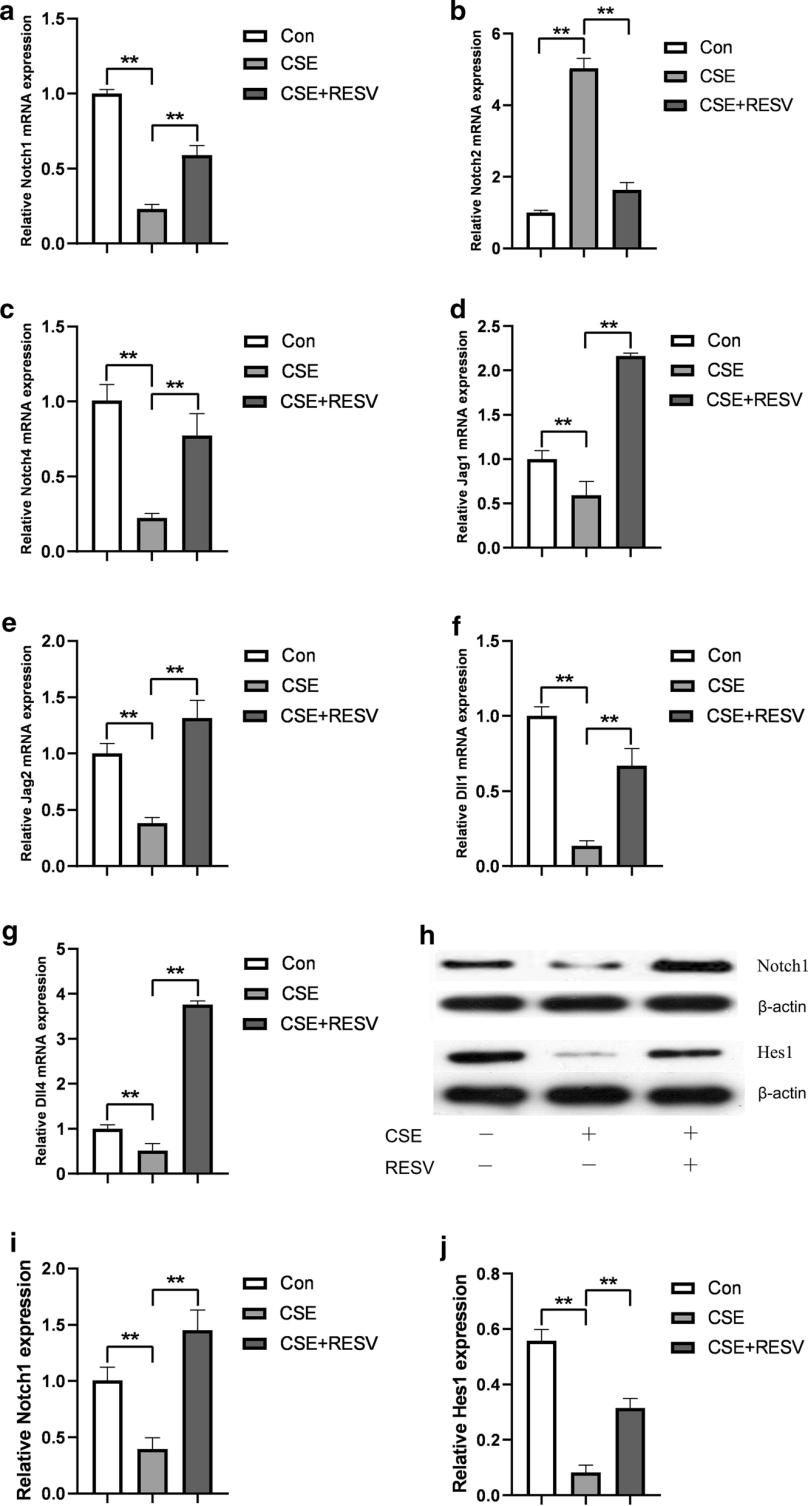
RESV restored the Notch1 signaling. mRNA expression of Notch receptors (Notch1, Nocth2, Notch4) and Notch ligands (Jag1, Jag2, Dll1, Dll4) were detected by qPCR (**a**, **b**, **c**, **d**, **e**, **f**, **g**). Notch1 and Hes1 protein level was detected by Western blot. β-Actin was used as loading control (**h**). Bar charts show the quantification of Notch1 (**i**) and Hes1 (**j**). Values are presented as means ± SD. *P < 0.05, **P < 0.01 compared between the marked groups

To elucidate the molecular mechanisms underlying the protective effect of RESV on endothelial apoptosis, we further analyzed the expression of the Notch signaling pathway-related genes. We have previously confirmed that Notch3 was mainly expressed in adult arterial vascular smooth muscle cells, rather than endothelial cells [2]. Thus, here we detected the other three Notch receptors and Notch ligands. The mRNA expression levels of Notch receptors and ligands were detected in HUVECs treated with CSE in the presence or absence of RESV. As shown in Fig. 3a–c, CSE decreased the mRNA expression of Notch1 and Notch4, while increased the expression of Notch2, which was in accordance with our previous study [2]. In addition, the mRNA expression of Notch ligands, Jag1/2 and Dll1/4, were also decreased by CSE (Fig. 3d–g). RESV pretreatment partly restored the mRNA levels of Notch receptors and ligands (Fig. 3a–g). The expression level of Notch1 protein was consistent with mRNA (Fig. 3h–i). Moreover, we detected the expression of Notch1 target gene, Hes1. The results showed a decrease in Hes1 expression in HUVECs that exposed to CSE. After cotreated with RESV and CSE, the expression level of Hes1 was partly restored (Fig. 3h, j). These results suggest a potential role of Notch signaling in the protective effect of RESV.

### RESV induced autophagy and reduced apoptosis in HUVECs in a Notch1-dependent manner

**Fig. 4:**
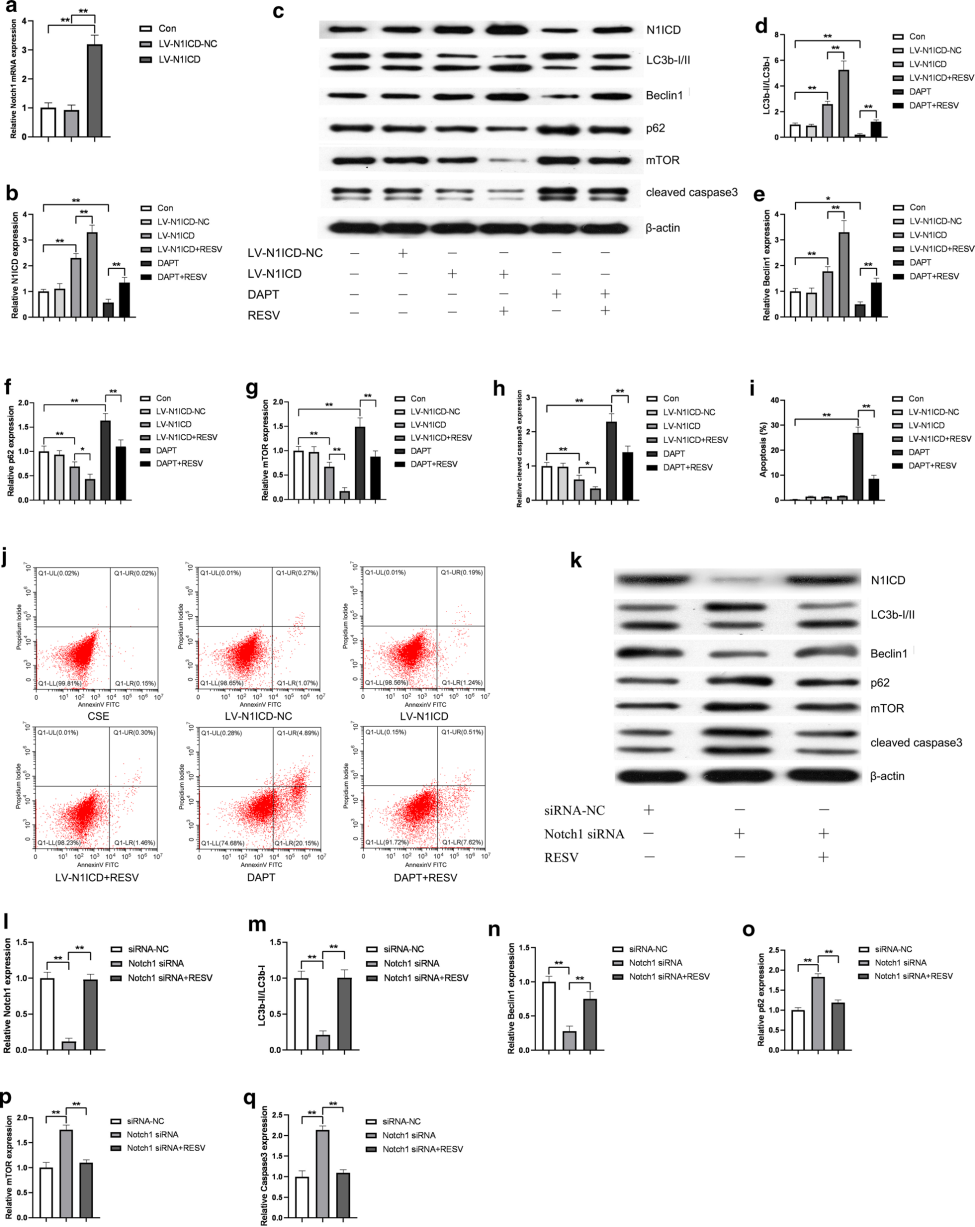
RESV stimulated autophagy and alleviated apoptosis through activating Notch1 signaling in CSE-treated HUVECs. N1ICD was overexpressed by LV-N1ICD transfection or suppressed by DAPT or siRNA in HUVECs as described in the Methods section. Notch1 mRNA expression was detected by qPCR (**a**). N1ICD protein, autophagy related proteins (LC3b-I/II, Beclin1, p62, mTOR) and apoptotic protein (cleaved caspase3) were detected by Western blot. β-Actin was used as loading control (**c**, **k**). Bar charts show the quantification of N1ICD, Beclin1, LC3B-II/LC3b-I, P62, mTOR and cleaved caspase3. Values are presented as means ± SD (**b**, **d**, **e**, **f**, **g**, **h**, **l**, **m**, **n**, **o**, **p**, **q**). Cell apoptosis was detected by flow cytometry (**j**). Statistical analysis was calculated (**i**). *P < 0.05, **P < 0.01 compared between the marked groups

We further investigated whether Notch1 is involved in the protective effect of resveratrol against CSE-induced endothelial apoptosis. Following the transfection and intervention of HUVECs, we evaluated changes in the expression level of autophagic and apoptotic markers. As shown in Fig. 4a–c, after N1ICD transfection, the mRNA expression of Notch1 and the protein level of N1ICD in the N1ICD lentivirus (LV-N1ICD) group was significant increased than that in the vector lentivirus (LV-N1ICD-NC) group (P < 0.05). These results indicated that the cells with N1ICD overexpression were successfully constructed. As expected, N1ICD overexpression increased the expression of LC3B-II and Beclin1, and decreased p62 and mTOR expression. Meanwhile, the cell apoptosis was also reduced (Fig. 4c–g).

RESV reversed the decrease in N1ICD expression by DAPT in HUVECs (Fig. 4b, c). After pretreated with RESV, the inhibition of LC3B-II and Beclin1 by DAPT were restored (Fig. 4c–e), and the expression levels of p62 and mTOR decreased (Fig. 4c, f, g). Moreover, pretreated with RESV yielded a protective effect from apoptosis. DAPT led to a significant induction of cleaved caspase3 levels and apoptosis rate. RESV rescued the increased apoptotic protein expression in HUVECs treated with DAPT (Fig. 4c, h), and resulted in a corresponding decrease in apoptosis rate (Fig. 4i, j).

To further demonstrate the effects of Notch1 on the protective process of RESV, Notch1 siRNA was used to knockdown Notch1 expression in HUVECs. The efficiency of siRNA-mediated knockdown of Notch1 was confirmed by Western blot (Fig. 4k, l). We detected that the effect of Notch1 siRNA transfection on cell autophagy and apoptosis was consistence with DAPT (Fig. 4k, m–q). RESV promoted autophagy and relieved apoptosis via upregulating Notch1 expression. These results demonstrated that RESV protected HUVECs from apoptosis by activating Notch1 signaling-dependent autophagy pathway.

### Perturbations of Notch1 or autophagy induced HUVECs apoptosis

**Fig. 5:**
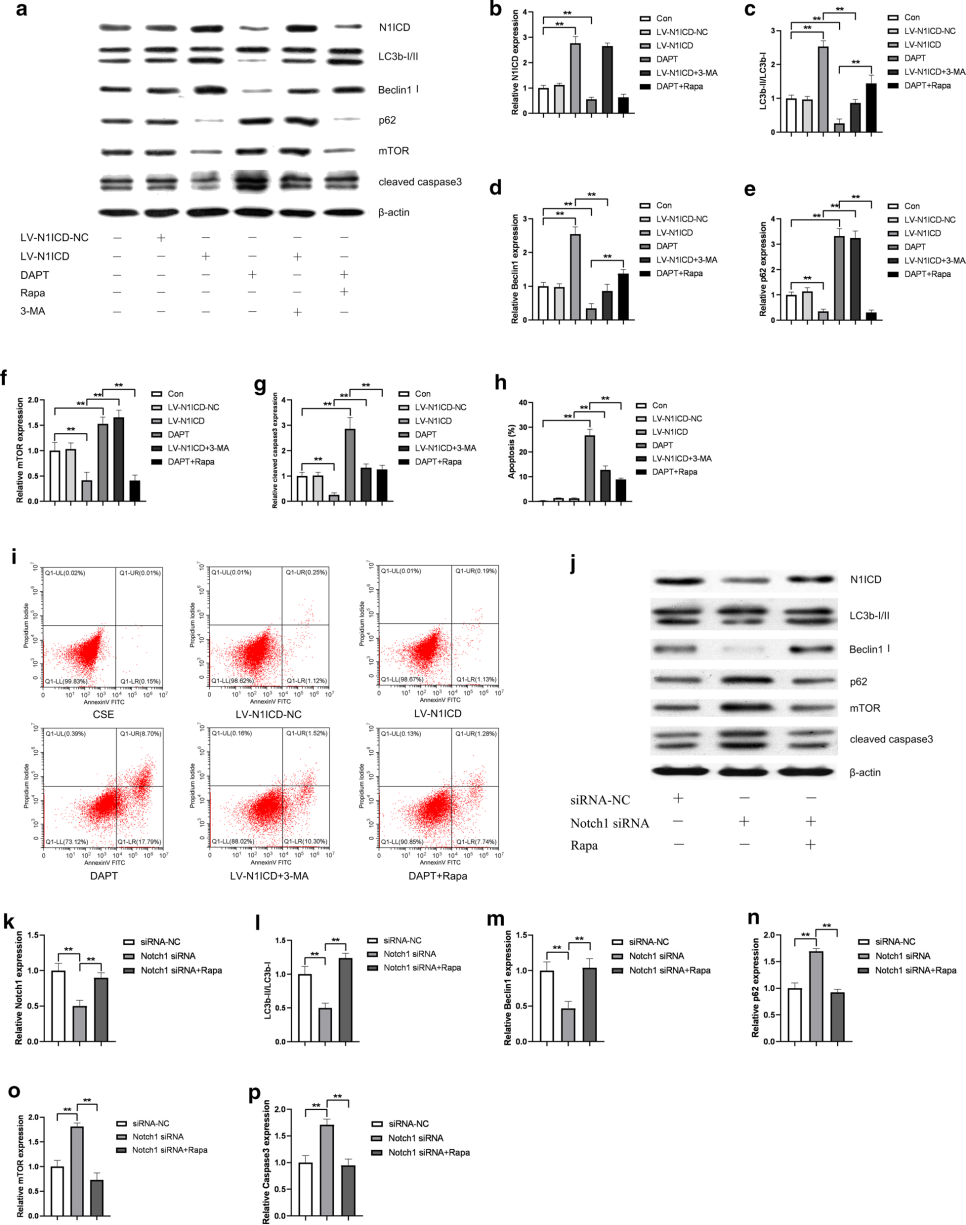
Notch1 protect HUVECs from CSE-induced apoptosis via activating autophagy. N1ICD protein, autophagy related proteins (LC3b-I/II, Beclin1, p62, mTOR) and apoptotic protein (cleaved caspase3) were detected by Western blot. β-Actin was used as loading control (**a**, **j**). Bar charts show the quantification of N1ICD, LC3B-II/LC3b-I, Beclin1, P62, mTOR and cleaved caspase3. Values are presented as means ± SD (**b**–**p**). Cell apoptosis was detected by flow cytometry (**i**). Statistical analysis was calculated (**h**). *P < 0.05, **P < 0.01 compared between the marked groups

In the previous study, we have confirmed a protective role of Notch1 in CSE-induced endothelial apoptosis [2]. Here, we assumed that Notch1 signaling might protect HUVECs from apoptosis via activating autophagy. To prove this hypothesis, we altered autophagy and Notch1 expression at the same time. We used LV-N1ICD and LV-N1ICD-NC to establish overexpression of Notch1 in HUVECs. Meanwhile, we interfered Notch1 expression by DAPT or Notch1 siRNA. As shown in Fig. 5, we found that LV-N1ICD effectively promoted the expression of Notch1 in HUVECs compared with vector control, while DAPT or Notch1 siRNA treatment significantly suppressed the Notch1 expression levels (Fig. 5a, b, j, k). The expression of LC3B-II and Beclin1 were increased in cells transfected with LV-N1ICD, and further increased in cells following resveratrol treatment (Fig. 5a, c, d). Simultaneously, the expression of p62 and mTOR decreased (Fig. 5a, e, f). On the contrary, DAPT or Notch1 siRNA had an opposite effect on the expression levels of autophagic markers (Fig. 5a, c–f, j, l-o). These results suggested a promoting role of Notch1 in autophagy. However, compared to those without 3-MA or rapamycin, no change in N1ICD protein expression was found in cells treated with them, suggesting Notch1 to be an upstream regulator of mTOR signaling. Then, we detected the apoptosis level by Western blot and flow cytometry. In accordance with our previous study, Notch1 overexpression reduced the apoptosis of HUVECs, while the inhibition of Notch1 increased the cell apoptosis. 3-MA treatment blocked the protective effect of Notch1 on apoptosis, while rapamycin restored the cell apoptosis induced by DAPT or Notch1 siRNA (Fig. 5a, g-i, j, p). In general, these data indicated that Notch1 protect apoptosis via activating autophagy in HUVECs.

## Discussion

In the present study, we provide evidence that RESV can suppress CSE- induced endothelial apoptosis via autophagy induction. RESV treatment also resulted in an increased expression of active Notch1 in HUVECs. DAPT or Notch1 siRNA significantly blocked Notch-1 activation and increased apoptotic protein expression, which can be partially reversed by RESV. Furthermore, the protective autophagy induced by RESV in HUVECs depends on a Notch1 signaling. Taken together, these data suggest that restoration of Notch1 signaling and induction of autophagy play critical roles in the protective effect of resveratrol on CSE- induced endothelial apoptosis.

Endothelial cell apoptosis is significantly elevated in the lungs of human smokers with COPD [5, 16], and mice with emphysema caused by CS exposure [7, 8], as well as in CSE treated endothelial cells in vitro [2–4, 6]. Consistent with previous studies, the present study further confirmed the promoting effect of CSE on the apoptosis of HUVECs. CS-induced endothelial dysfunction/injury have been linked to the pulmonary lesions in COPD and systemic co-morbidities including atherosclerosis, pulmonary hypertension, and chronic renal injury [17], suggesting that CS can cause multisystem diseases, or at least endothelium-associated diseases, through endothelial apoptosis. It has been shown that direct induction of endothelial cell apoptosis using an endothelial cell-specific peptide results in emphysematous lung destruction. while inhibition of endothelial apoptosis effectively prevented the emphysematous changes [7, 8]. These results highlight a crucial role of endothelial apoptosis in the pathogenesis of CS-related diseases. Therefore, preventing endothelial cell from CS-induced apoptosis and strengthening the endothelial barrier may be an effective therapeutic strategy.

RESV is a polyphenol naturally found in a variety of plant species such as grapes, apples, berries, and peanuts. It is well known to have beneficial effects, including anti-inflammatory, anti-oxidative stress and anti-apoptosis [9, 18]. The anti-apoptotic effects of RESV have been widely investigated in multiple organs, including lungs. Ceramide was reported to be up-regulated in the lungs of COPD patients and CS exposed mice, and was shown to be associated with the increased apoptosis of bronchial epithelial cells [19]. RESV protects bronchial epithelial cells from CS-induced apoptosis in vitro and in mouse models through attenuating the accumulation of ceramide [20]. As a natural activator of SIRT1, RESV is capable of protecting bronchial epithelial cells against CSE-induced apoptosis through activating SIRT1 [21]. The results indicated the anti-apoptotic function of RESV in bronchial epithelial cells in the condition of CS exposure. However, few studies investigated whether it has a similar protective effect on CSE-induced endothelial cell apoptosis. In the present study, we have strong evidence that resveratrol attenuates CSE- induced HUVEC apoptosis. Pretreatment with RESV remarkably attenuates the expression of cleaved caspase-3, an indicator for cell apoptosis, in the presence of CSE. Moreover, its protective effect against HUVEC apoptosis under CSE is associated with autophagy. Autophagy is an essential intracellular process responsible for the selective degradation of pathogens, damaged organelles and proteins that cannot be degraded by the proteasome, to support protein and organellar homeostasis, as well as recycling of biomolecular resources, to maintain energy homeostasis and cell survival [22]. Emerging evidence indicates that RESV attenuates endothelial oxidative injury in HUVECs by inducing autophagy [11, 23]. Here, in the present study we found that blocking autophagy with 3-MA aggravated the apoptosis by upregulating caspase3 expression, while inducing autophagy with rapamycin relieved the apoptosis, indicating that autophagy confers the ability to protect endothelial cells from CSE- induced apoptosis. Moreover, we observed a promoting effect of RESV on autophagy by inhibiting mTOR signaling pathway. Cells treated with RESV showed a decrease in the expression of mTOR protein, resulting in an upregulation in Beclin1 and LC3b-II expression, with a decrease in p62 levels compared to those without RESV treatment. However, the exact underlying mechanism remains unclear and requires further investigation.

Notch1 receptor protein is one of the four single-pass transmembrane receptors in Notch system, which controls cell proliferation, differentiation, and apoptosis. Notch signal activation involves the ligand-receptor interaction from the surface of two neighbouring cells, which triggers two successive proteolytic cleavages and release of the NICD from the full-length heteromeric receptor. Then, NICD travels to the nucleus where it interacts with the DNA-binding protein and converts it into a potent transcriptional activator [24]. Our previous studies have demonstrated that Notch-1 was downregulated in lung tissue of COPD compared to those of non-smokers [2]. Furthermore, overexpressing Notch-1 has been shown to alleviate CSE mediated endothelial apoptosis [2, 3]. Recently, several studies have shown that Notch1 is involved in the autophagic process [15, 25]. We speculate that autophagy may be related to the protective effect of Notch1 against endothelial apoptosis. We found that Notch1 expression was decreased after CSE exposure in consistent with the previous studies. Notch1 inhibition leads to an increase in the expression of mTOR and a decrease Beclin1 and LC3b-II expression accompanied with increased p62 levels, as well as an increase in endothelial apoptosis. Overexpression of Notch1 induced autophagy by inhibiting mTOR signaling, meanwhile attenuated the HUVECs apoptosis in endothelial cells. Furthermore, rapamycin reversed the cell apoptosis resulting from the inhibition of Notch1, while 3-MA blocked the anti-apoptotic effect of Notch1 signaling. These results suggested that Notch1 heavily inhibits cell apoptosis of HUVECs by promoting mTOR-mediated autophagy.

Recent years, RESV has received increasing attention for its role in regulating the Notch signaling. However, conflicting results of RESV effects on Notch1 signaling were reported that RESV can enhance or suppress Notch1 in a cell type dependent manner. Yu et al. [13] found that RESV inhibits cell growth and enhances redifferentiation in anaplastic thyroid carcinoma cells via activation of Notch1 signaling. Lin and his colleagues [26] detected that RESV restored the expression of p53 through activating Notch1 signaling, which lead to anti-proliferative and pro-apoptotic effects on glioblastoma cells. Different opinion was put forward by Zhong et al. [27], they found that RESV exerts inhibitory effects on human ovarian cancer cells by blocking Notch1 signaling. In this work, we examined what role RESV plays in HUVECs in the condition of CSE exposure. We observed that RESV treatment significantly restored the Notch1 protein levels suppressed by CSE. Furthermore, supplementation with RESV improves the cell apoptosis in HUVECs resulted from the inhibition of Notch1 signaling. Taken together, the results from the present study indicated that RESV elicits its protective effect against CSE-induced apoptosis through regulating Notch1-mediated autophagy.

## Conclusions

In summary, our study suggested that RESV pretreatment is associated with relieved CSE-induced endothelial apoptosis at least partly via promoting autophagy mediated by a Notch1-dependent mechanism. Our results provide insights into the possibility that Notch1 is targeted by RESV and suggest that the associated induced autophagy may have implications for improving CSE-induced endothelial apoptosis. These findings support the therapeutic potential of RESV for the prevention and treatment of CSE-related diseases, such as COPD.

## Data Availability

Essential datasets supporting the conclusion are included in this published article.

## Abbreviations

3-MA: 3-Methyladenine
COPD: Chronic obstructive pulmonary disease
CS: Cigarette smoke
CSE: Cigarette smoke extract
DAPT: γ-Secretase inhibitor
HUVEC: Human umbilical vein endothelial cell
mTOR: Mammalian target of rapamycin
NICD: Notch intracellular domain
Rapa: Rapamycin
RESV: : Resveratrol
